# Obesity and Resting Metabolic Rate Assessed by Indirect Calorimetry in Pediatric Patients from Northeastern Romania

**DOI:** 10.3390/diagnostics16020320

**Published:** 2026-01-19

**Authors:** Lorena Mihaela Manole, Elena Țarcă, Laura Otilia Boca, Mădălina Andreea Donos, Elena-Lia Spoială, Iulia Margasoiu, Otilia Elena Frăsinariu, Nicoleta Gabriela Ciobanu-Hașovschi, Viorel Țarcă, Laura Mihaela Trandafir

**Affiliations:** 1 Grigore T. Popa University of Medicine and Pharmacy, 700115 Iași, Romania; lorena.manole@umfiasi.ro (L.M.M.); laura.boca@umfiasi.ro (L.O.B.); elena-lia.spoiala@umfiasi.ro (E.-L.S.); frasinariu.otilia@umfiasi.ro (O.E.F.); hasovschi.gabriela-nicoleta@d.umfiasi.ro (N.G.C.-H.); laura.trandafir@umfiasi.ro (L.M.T.); 2 Saint Mary Emergency Children Hospital, 700309 Iași, Romania; 3 Nutriwise by Iulia Margasoiu, 707410 Iași, Romania; 4 “Socola” Institute of Psychiatry, 700282 Iași, Romania; 5 Faculty of Dental Medicine, Apollonia University, 700511 Iași, Romania; viorel.tarca@univapollonia.ro

**Keywords:** obesity, indirect calorimetry, resting metabolic rate, nutritional therapy, children, adolescents

## Abstract

Pediatric obesity is a growing public health concern, significantly increasing the risk of metabolic and cardiovascular comorbidities. **Background/Objectives**: This study aims to explore the burden of obesity, its associated comorbidities, and resting metabolic rate (RMR) assessed by indirect calorimetry among children and adolescents in a cohort of 223 participants from Nord-East of Romania. **Methods**: A cross-sectional study was conducted among 223 children and adolescents (aged 4–18 years) who were diagnosed with obesity at Saint Mary Emergency Children’s Hospital Iași. Anthropometric measurements, clinical assessment, and biochemical parameters were recorded. RMR was measured by indirect calorimetry, using the Fitmate Pro Metabolic Technology (Cosmed, Rome, Italy), under a stable environment for 15 min, following a fasting period of minimum 6–8 h. Data were analyzed using SPSS 22.0, applying descriptive statistics and Pearson correlations. **Results**: A total of 223 participants were included in the analysis, with a mean age of 12.03 ± 3.32 years (range 4–17 years) and a mean body mass index (BMI) of 31.21 ± 5.84 kg/m^2^. The average RMR was 1687.5 ± 425.5 kcal/day, with higher values in males compared with females. RMR showed significant positive correlations with age (r = 0.60), BMI (r = 0.51), waist circumference (r = 0.67), and fat mass measured with a three-site formula technique (r = 0.51) and systolic (r = 0.45) and diastolic blood pressure (r = 0.19), all with *p* < 0.001. A weak inverse correlation was observed between RMR and the fitness index (r = −0.24, *p* < 0.001), indicating an association between lower fitness scores and higher RMR values. RMR showed no significant correlation with fasting glucose or lipid levels, indicating that metabolic rate was more influenced by body composition than by biochemical markers. **Conclusions**: Pediatric obesity is strongly linked to multiple comorbidities, emphasizing the need for early detection and targeted interventions. Higher BMI and central adiposity were associated with increased RMR. Indirect calorimetry provides valuable insights into the metabolic profile of children with obesity and can inform individualized management strategies.

## 1. Introduction

Obesity is a complex and multifactorial condition, ranking among the most common pathologies globally and one of the most pressing public health challenges of our time for children [1,2,3]. Its prevalence continues to rise in both pediatric and adult populations, posing a significant risk of comorbidities and resulting in substantial social and economic impacts [4].

The global burden has continued to rise across the life course; from 1990 to 2022, obesity approximately doubled in adults and quadrupled among those aged 5–19 years [2,5]. In Romania, estimates vary by data source and method. Self-reported height and weight data from 2022 (European Health Interview Survey) indicate that about one in four 15-year-olds in Romania are overweight, with higher rates in boys than girls [6]. For younger, primary school children, WHO’s European Childhood Obesity Surveillance Initiative (COSI) synthesis estimated severe obesity at ~5.1% by WHO cutoffs, underscoring a measurable burden in life [7].

Obesity is defined by the World Health Organization as abnormal or excessive fat accumulation that impairs health [3]. In adults, this corresponds to body mass index (BMI) ≥ 30 kg/m^2^, but for school-age children and adolescents (5–19 years), classification relies on BMI-for-age z-scores: overweight > +1 standard deviation (SD) and obesity > +2 SD. Also, it is necessary to complement clinical assessment with waist circumference and related measures [2,3].

Obesity in children can profoundly influence overall well-being, heightening the risk of a broad spectrum of metabolic and cardiovascular complications [8,9], including insulin resistance, type 2 diabetes mellitus (T2DM), hypertension, dyslipidemia, and metabolic dysfunction-associated steatotic liver disease (MASLD) [2,10,11,12]. Beyond their immediate health effects, these conditions significantly elevate the risk of adult morbidity and mortality [13]. In addition, obesity is associated with psychological difficulties, including low self-esteem, depression, and social stigma, which further amplify the overall disease burden [14]. Current and precise methods for assessing obesity in children and adolescents are crucial for early prevention of these comorbidities. Also, there is an urgent need to better understand the metabolic mechanisms that govern energy balance in children.

The management of obesity in children and adolescents is inherently complex and requires a multidisciplinary, age-appropriate strategy that integrates nutrition therapy, physical activity, and behavioral interventions [15]. In order to reduce excess weight, a hypocaloric diet remains the main fundamental part, but it should be individualized to the child’s stage of growth to achieve gradual, appropriate weight loss. Also, it is important to improve body composition, metabolic risk markers, and quality of life [16,17,18].

Effective prevention and treatment of this chronic disease require precise characterization of energy balance. This will enable the design of a tailored diet after determining each patient’s energy requirements, so that an appropriate caloric deficit can be prescribed [19]. The correct prescriptions depend on accurately characterizing total energy expenditure (TEE), which represents the 24 h energy cost of the body and comprises three main components: diet-induced thermogenesis; resting metabolic rate (RMR), often used interchangeably in the literature with resting energy expenditure (REE); and energy expended in physical activity (or activity thermogenesis) [20,21,22]. Indirect calorimetry (IC) provides a non-invasive assessment of energy metabolism by quantifying oxygen consumption and carbon dioxide production to estimate RMR [23]. IC is considered a reference method for assessing RMR, which represents the energy required to sustain vital functions of the body at rest [24].

Effective assessment of the RMR is essential in healthy obese and overweight young people because the interventions associated with weight loss may potentially have important implications in long-term weight management and prevention of the comorbidities [19].

Although obesity is a major pediatric health problem, relatively few studies have examined RMR measured by IC in children and adolescents, particularly in relation to accurate energy requirement estimation and its potential role in individualized dietary planning. The aim of this original retrospective study is to evaluate the burden of obesity and comorbidities and investigate the relationship between RMR and anthropometric, as well as biochemical, markers in children and adolescents from northeastern Romania, in order to improve clinical assessment and guide personalized interventions.

## 2. Materials and Methods

### 2.1. Participants

The patient’s data were retrospectively reviewed from the units from “Sfânta Maria” Emergency Children’s Hospital in Iași, a city of Nord-East of Romania. The search targeted individuals with obesity, as determined by the anthropometric measurements during their initial visit, prior to any structured weight loss intervention. These patients had also RMR assessment using IC during their visit, which is standard practice in the nutrition department as part of their nutritional assessment, biochemical markers, and radiological investigations interpreted against standard reference ranges. Enrollment began on the date of Ethics Committee approval and continued through the predefined study period. The study complied with the Declaration of Helsinki. Written informed consent was obtained from the parent or legal guardian, and age-appropriate assent was obtained from participants.

Inclusion criteria were as follows:

- Patients aged 4–18 years at enrollment;
- Patients with newly diagnosed obesity defined by age- and sex-specific BMI using a single, pre-specified standard (WHO growth reference for children—BMI-for-age > +2 SD);
- Patients with no prior structured diet, weight-loss program, and no prior pharmacologic treatment for weight reduction;
- Patients with written informed consent from a parent/legal guardian before any procedure;
- Patients with adherence to test conditions for RMR, which were
  (1) Overnight fast (minimum 8 h), morning testing preferred;
  (2) No moderate–vigorous exercise for 24 h prior;
  (3) No caffeine or stimulant beverages for 12 h prior;
  (4) Resting quietly (supine position) for 15 min in a thermoneutral room before measurement;
  (5) Ability to tolerate canopy hood and remain still during the test.

Exclusion criteria:

- Patients with normal weight or underweight status, defined as BMI-for-age z-score < +1 SD;
- Patients with medical conditions such as
  (1) acute illness, fever, or infection within the past 2 weeks;
  (2) uncontrolled endocrine/metabolic disease (hypo-/hyperthyroidism, Cushing syndrome), decompensated diabetes, or other conditions that can substantially alter RMR (e.g., severe anemia, active malignancy);
  (3) severe psychiatric or neurodevelopmental conditions that preclude cooperation with investigator or with testing;
- Patients with chronic therapies (e.g., systemic glucocorticoids, chemotherapy, sympathomimetics or other substances known to materially affect metabolic rate);
- Refusal or withdrawal of informed consent;
- Known or suspected pregnancy in post-menarchal female participants.

### 2.2. Ethical Approval

The study has been approved and declared as ethically appropriate by the Ethics Committee of Saint Maria Emergency Children’s Hospital, Iasi, Romania (approval date: 26 Octomber 2023), and subsequently by the Research and Ethics Scientific Committee 412/11.03.2024 released by Grigore T. Popa University of Medicine and Farmacy Iasi, Romania (approved date: 11 March 2024). Screening of obesity was performed with the consent of the parents of the subjects.

### 2.3. Study Design

**Figure 1:**
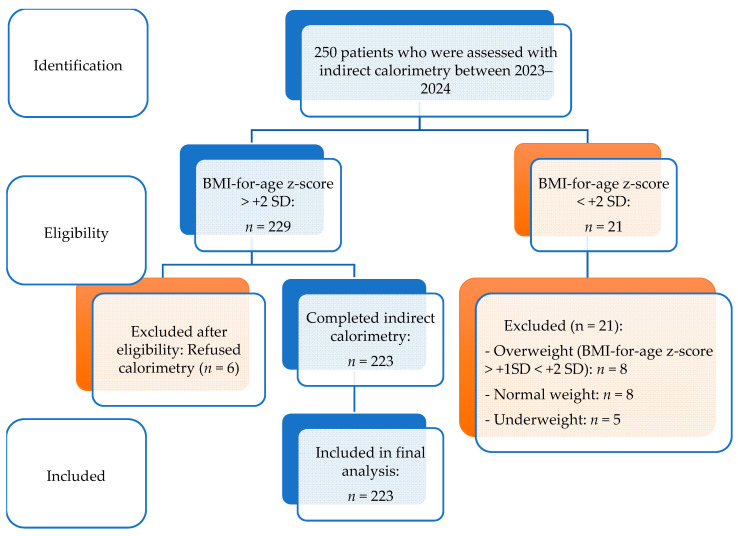
Flow diagram of participant selection and inclusion. BMI categories were defined according to WHO BMI-for-age z-score criteria [3].

This study was conducted at the Saint Maria Emergency Children’s Hospital in Iași, Romania, and was designed as a descriptive, cross-sectional analysis. Following a comprehensive evaluation of the patient database, data were collected for a cohort of 223 children and adolescents who met the specified inclusion criteria. This sample size was considered adequate given the aims of the study and the characteristics of the investigated population. Moreover, due to its observational design, the study provides valuable preliminary insights for future research and allows for the formulation of initial conclusions aligned with the study objectives. The participant selection process is illustrated in Figure 1.

The collected data included the following: unique participant IDs, sex, age, weight, height, BMI, fat mass (FM), fat mass percentage (FM%), lean mass (LM), and RMR measured through IC, as well as relevant biochemical parameters. Following data collection, statistical analyses were performed to examine correlations between variables and to address the research questions of the study.

A total of 223 children and adolescents (108 girls and 115 boys) aged between 4 and 18 years (mean age 12.03 ± 3.31 years) diagnosed with obesity were included in the study. The primary aim was to individualize energy prescriptions by quantifying RMR and to monitor clinical, biochemical, and imaging responses over time, with the ultimate goal of achieving and maintaining a healthy weight trajectory.

### 2.4. Data Collection

The patient’s data were retrospectively reviewed from the medical records to evaluate the following: clinical examination, biological and imaging investigations, indirect calorimetry, and nutritional consultation. Anthropometric measurements, like height, weight, BMI, and waist circumference, were recorded following standardized protocols. Height and weight were measured with participants wearing light clothing and no shoes. BMI-for-age percentile and z-score were generated using the WHO Anthro and WHO AnthroPlus software version 1.0.4 (World Health Organization, Geneva), referencing the WHO 2007 growth standard [25]. FM, FM%, and LM were evaluated using skin fold thickness measurements introduced in the calorimeter. The device also generated the waist-to-hip ratio and fitness index based on the recorded circumferences and data.

Systolic and diastolic blood pressure (BP) was measured using a manual sphygmomanometer, with elevated values classified according to pediatric hypertension guidelines. BP measurements were interpreted according to age-, sex-, and height-specific reference values. Systolic and diastolic BP percentiles were obtained using the “Blood Pressure Percentiles, 0 to 17 years (2017 standard)” clinical calculator, available in the MSD Manuals Professional Edition (Merck & Co., Inc., Rahway, NJ, USA). Values were classified as normal, elevated, or hypertensive according to the 2017 American Academy of Pediatrics [26,27].

Fasting blood samples were collected to assess key parameters such as glucose metabolism (HOMA-IR), lipid profiles, and inflammatory markers. These variables play crucial roles in accurate evaluations and assist clinicians in determining appropriate therapeutic strategies for weight management.

**Figure 2:**
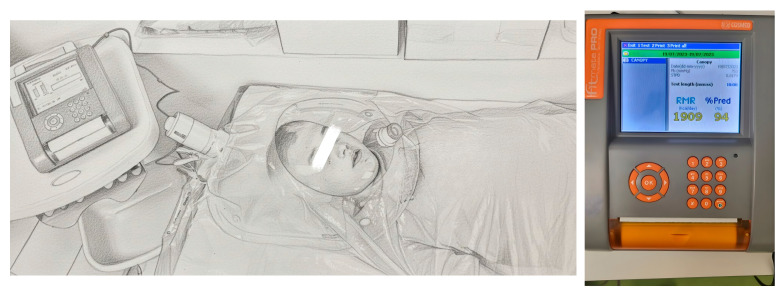
Schematic illustration of the patient setup for indirect calorimetry assessment and a photograph of the metabolic cart used for resting metabolic rate measurement.

RMR was measured through IC, using a portable metabolic calorimeter, the Fitmate Pro Metabolic Technology, COSMED, Rome, Italy, with a ventilated canopy (hood) system (Figure 2).

The measurements were performed in the morning, following an overnight fasting period of about 8–12 h, with participants resting in a supine position and minimizing movement during the procedure. All tests were conducted in a thermoneutral room, with a quiet, stable environment for 15 min. The first 5 min of data acquisition were discarded. The patients were refraining from vigorous physical activity, caffeine, or stimulant beverages for at least 24 h prior to testing. To assess RMR, patients were asked to lay back on the hospital bed and were instructed to lie still, avoid speaking, and not fall asleep during the evaluation. Oxygen consumption (VO_2_) was averaged over a steady-state period of 10 min, defined as <10% variability in VO_2_ and respiratory parameters. RMR was automatically calculated by the device using the abbreviated Weir equation:

RMR = [3.9 (VO_2_) + 1.1 (VCO_2_)] × 1.44.

The respiratory quotient (RQ), defined as VCO_2_/VO_2_, was used to support interpretation of metabolic substrate utilization and assess data quality. Steady state was defined as a minimum of 5 consecutive minutes with <10% variation in VO_2_ and VCO_2_ and <5% variation in RQ. Total measurement duration was 15 min to ensure stable and valid data. RMR measurements were performed using the automatic test execution mode of the Fitmate system, which includes automatic detections of test start and end, as well as automatic selection of a stable averaging interval. Assessments were conducted once per participant, in accordance with routine clinical protocol, without repeated measurements or within-subject variability analyses being performed.

The Fitmate Pro device was prepared and operated in accordance with the manufacturer’s recommendations. Device calibration and sensor checks were performed following standard internal procedures specified by the manufacturer before testing sessions. The device was calibrated according to the manufacturer’s instructions using a 3-L syringe.

After completion of the RMR test and manual entry of the anthropometric data, the calorimetry device automatically calculated and provided the waist-to-hip score, FM%, and fitness index as part of the output parameters.

Body composition was also assessed using the Fitmate Pro system (COSMED, Rome, Italy). Skinfold thickness measurements were obtained according to the manufacturer’s instructions and entered into the device software, which automatically calculated body composition parameters using proprietary algorithms. The three-site skinfold protocol included measurements at the upper thoracic region in boys, the upper arm region in girls, and the abdominal and thigh regions in all participants, as specified by the device guidelines. All measurements were performed by the same two trained investigators throughout the study, both instructed with the device operation and adhering to standardized manufacturer procedures.

The “fitness index” represents a composite score generated by the Fitmate software version 2.4 (build 29; COSMED S.R.L, Rome, Italy), based on internally integrated anthropometric and physiological inputs. According to the manufacturer’s documentation, the fitness index integrates multiple measured and derived parameters, including body mass index, waist-to-hip ratio, resting heart rate, systolic and diastolic blood pressure, body fat percentage, muscular strength tests, flexibility, and estimated VO_2_ per kilogram. The exact weighting and mathematical algorithm used to compute the final score are not publicly disclosed by the manufacturer. Therefore, the fitness index was analyzed as a descriptive, device-generated indicator and not as a validated standalone measure of physical fitness. This index is provided by the device as an exploratory parameter and does not correspond to an independent, externally validated physical fitness assessment tool.

### 2.5. Statistical Analysis

Data analysis was conducted using SPSS software (version 22.0; IBM SPSS Corp., Armonk, NY, USA), the R open-source statistical environment (version 3.4; R Core Team, 2017, Vienna, Austria), and Microsoft Excel (Microsoft Corp., Redmond, WA, USA). Continuous variables were summarized as mean ± SD, median, and variance, depending on the underlying distribution, while categorical variables were presented as counts and percentages. Additionally, age distribution was examined in relation to sex.

In R, group comparisons for continuous variables were performed using the Welch two-sample *t*-test, chosen for its robustness in the context of unequal variances between groups. Categorical variables were assessed using Pearson’s Chi-squared test with Yates’ continuity correction, and proportions were compared using the one-sample proportions test with continuity correction.

Associations between RMR and metabolic parameters were examined using Pearson’s correlation coefficient, computed in both R and SPSS. Furthermore, regression plots were generated to evaluate relationships between age and the other parameters, including the association between RMR and BMI. In SPSS, additional procedures included the generation of linear regression plots, computation of Pearson correlations between RMR and metabolic indicators, and descriptive statistics for continuous variables, as well as analyses of medical parameters stratified by sex. Assumptions of normality and homoscedasticity were evaluated prior to selecting parametric tests. Statistical significance was set at *p* < 0.05 for all analyses. Given the descriptive and exploratory nature of the study and the absence of a single predefined primary hypothesis, no formal adjustment for multiple comparisons was applied. All *p*-values are reported to allow transparent interpretation of statistical uncertainty.

## 3. Results

**Figure 3:**
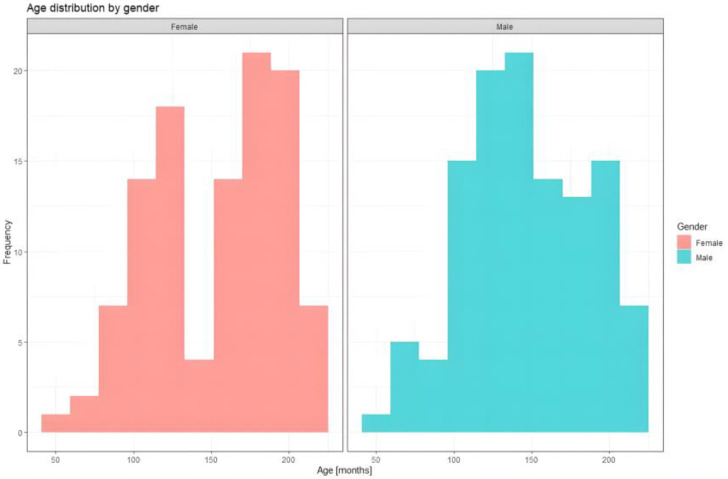
Distribution of the study cohort by sex (female/male).

A total of 223 participants were included in the analysis, with a mean age of 12.03 ± 3.32 years (range 4–17 years), corresponding to 149.65 ± 39.96 months. The sex distribution was approximately 48.4% females and 51.57% males (Figure 3). Most participants originated from urban areas (51.12%). Anthropometric evaluation showed a mean BMI was 31.21 ± 5.84 kg/m^2^, while mean waist circumference was 91.63 ± 13.91 cm (range 52–140 cm). More descriptive statistics are shown in Table 1.

**Figure 4:**
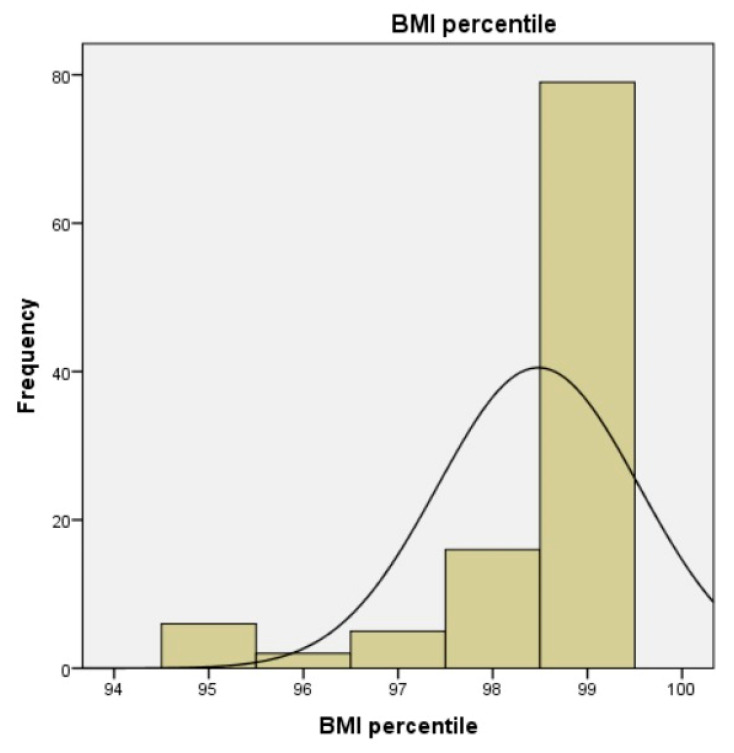
Distribution of BMI percentiles in the study cohort, highlighting the number of children with elevated values.

The mean BMI Z-score was 3.2 ± 1.01, confirming the diagnosis of obesity for the subjects, and according to the BMI percentiles, most participants were at or above the 99th BMI percentile, indicating severe obesity according to pediatric growth standards (Figure 4). Analysis of BMI z-scores showed that 97 participants were classified between +2.0 and 2.99 SD, and 78 patients were classified between +3.0 and +3.99 SD, with 37 individuals above +4 SD, while only 11 children had z-scores below +2.0 SD.

The mean RMR was 1687.53 ± 425.48 kcal/day, with values ranging from 846 to 3357 kcal/day. FM% presented a mean of 33.26 ± 5.37% (range 18.8–43.2%). Based on RMR values obtained by IC and using the age-adjusted classification thresholds provided by the device software, 72 participants (32.3%) were classified as having a slow metabolic rate, 139 (62.3%) were classified as having a normal metabolic rate, and 12 participants (5.4%) were classified as having a fast metabolic rate. Correspondingly, mean FM was 26.75 ± 10.21 kg. The waist-to-hip ratio was 1.27 ± 5.56, indicating considerable variability in fat distribution among participants and suggesting that a substantial subset of the cohort exhibited a central adiposity profile. A total of 185 patients (83%) had the waist circumference percentile over 90, which was interpreted according to the waist circumference percentile recommended by the International Diabetes Federation for the diagnosis of metabolic syndrome in children and adolescents [28,29]. The waist-to-height ratio (WHtR) presented a mean of 0.582 ± 0.065, and based on its values, a total of 207 participants had a WHtR ≥ 0.50, indicating central adiposity.

**Figure 5:**
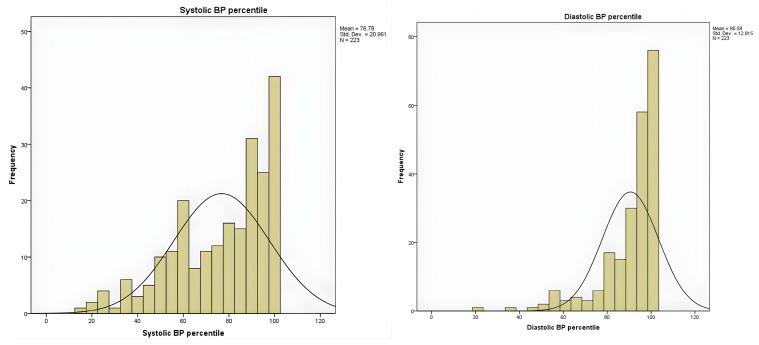
Distribution of systolic and diastolic blood pressure percentiles in the study cohort, highlighting the number of children with elevated values.

Systolic BP averaged 118.3 ± 14.75 mmHg and diastolic BP 81.68 ± 11.03 mmHg among the children and adolescents. Interpretation of blood pressure measurements was based on reference standards adjusted for age, sex, and height, from which systolic and diastolic percentiles were calculated. The distribution of BP percentiles showed a right-skewed pattern, with a mean of 76.78 ± 20.96 for systolic BP percentile. A considerable proportion of participants exhibited systolic BP in hypertensive ranges: 11.7% were above the 99th percentile, and 13.5% were between the 95 and 98th percentiles, indicating a high prevalence of elevated systolic readings in this cohort. Similarly, diastolic blood pressure percentiles were shifted toward higher values, with a mean of 90.58 ± 12.81 percentile for diastolic BP percentile (Figure 5). More than half of participants (57.4%) were at or above the 95th percentile, of which 34.1% exceeded the 99th percentile. Overall, the majority of children showed elevated diastolic blood pressure, suggesting a pattern consistent with obesity-related hypertension.

**Figure 6:**
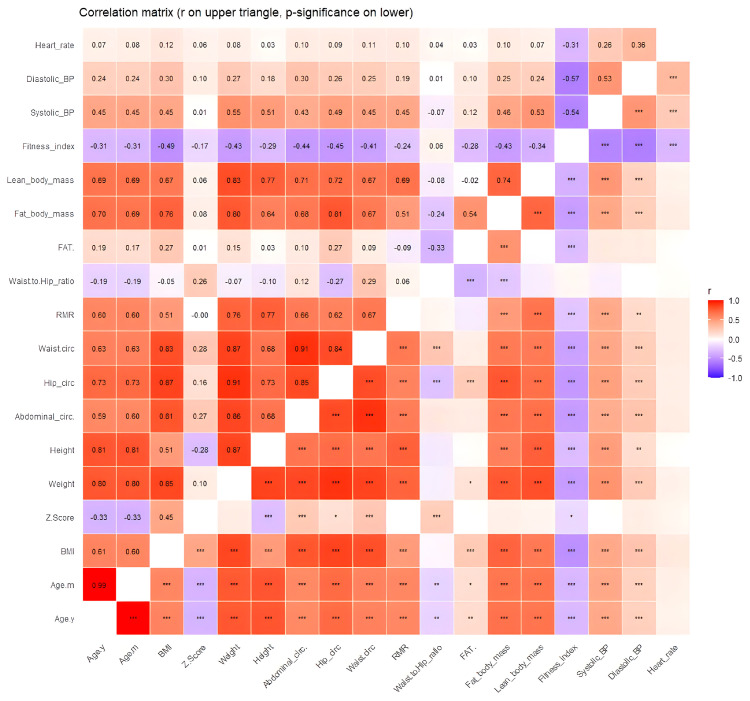
Correlations between RMR and anthropometric data (r on upper triangle, *p*-significance on lower triangle). * indicates *p* < 0.05, ** indicates *p* < 0.01, and *** indicates *p* < 0.001).

Correlation analyses revealed multiple statistically significant associations among the investigated variables; the full set of correlations is presented in Figure 6, while below, we report only those most relevant to the aims of this study.

Strong associations were also found with height (r = 0.77, *p* < 0.001), weight (r = 0.76, *p* < 0.001), BMI (r = 0.51, *p* < 0.001), and age (months/years, r = 0.60, *p* < 0.001). RMR demonstrated positive correlations with systolic (r = 0.45, *p* < 0.001) and diastolic BP (r = 0.19, *p* < 0.01), as well as with LM (r = 0.69, *p* < 0.001), FM (r = 0.51, *p* < 0.001), waist (r = 0.67, *p* < 0.001), hip (r = 0.62, *p* < 0.001), and abdominal circumference (r = 0.66, *p* < 0.001). RMR showed a weak but statistically significant inverse correlation with the fitness index (r = −0.24, *p* < 0.001). According to fitness index interpretation, 46.6% (104 patients) was interpreted as poor, and 19.3% (43 children) was interpreted as a very poor index. A percentage of 25.6% (57 individuals) had fair as interpretation, 18 patients had good, and just 1 individual had an excellent index. A weak correlation was observed between RMR and WHtR (r = 0.176, *p* = 0.008).

BMI displayed similarly robust correlations with age (years, r = 0.61; months, r = 0.60; both *p* < 0.001), weight (r = 0.85, *p* < 0.001), height (r = 0.51, *p* < 0.001), and BMI z-score (r = 0.45, *p* < 0.001). Significant positive associations were also observed with abdominal (r = 0.81, *p* < 0.001), hip (r = 0.87, *p* < 0.001), and waist circumference (r = 0.83, *p* < 0.001), as well as with FM (r = 0.76, *p* < 0.001), LM (r = 0.67, *p* < 0.001), FM% (r = 0.27, *p* < 0.001), and RMR (r = 0.51, *p* < 0.001). BMI also correlated positively with systolic (r = 0.45, *p* < 0.001) and diastolic BP (r = 0.30, *p* < 0.001) and negatively with the fitness index (r = −0.49, *p* < 0.001). A strong positive correlation was found between BMI and WHtR (r = 0.647, *p* < 0.001).

**Figure 7:**
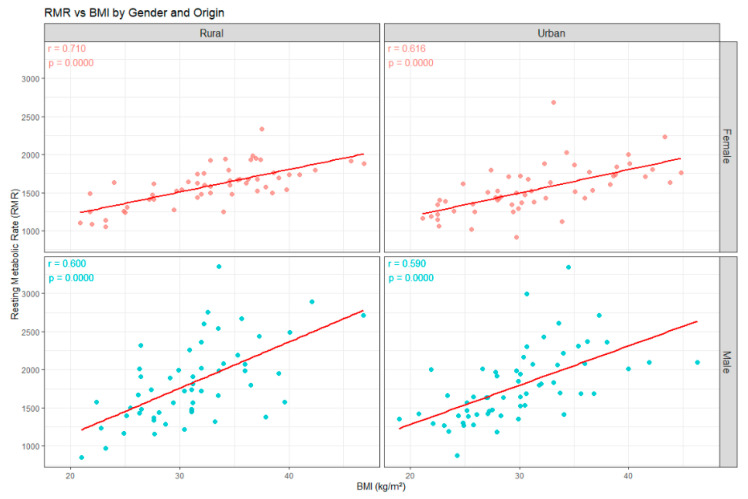
Correlation between RMR and BMI.

A positive and statistically significant correlation was observed between BMI and RMR across the study population and all subgroups, regardless of sex or area of residence (Figure 7). The scatterplot demonstrates an upward trend in RMR values with increasing BMI, consistent with the significant association identified in the correlation analysis. The strongest associations were observed in females (rural was r = 0.710, *p* < 0.001, and urban r = 0.616, *p* < 0.001), followed by males (rural was r = 0.600, *p* < 0.001, and urban r = 0.590, *p* < 0.001). These findings indicate that higher BMI is consistently associated with higher RMR. Notably, the correlations coefficients were consistently higher in rural compared with urban participants for both sexes. This may suggest potential contextual or lifestyle differences influencing the relationship between body mass and metabolic expenditure, although further investigation is needed to clarify the underlying mechanisms.

The boxplots positioned along the axes illustrate the variability of both parameters, confirming a wide dispersion of RMR, particularly at higher BMI values, with several high-energy outliers. These findings support the relationship between increased adiposity and elevated resting energy demands in children and adolescents with excess weight.

**Figure 8:**
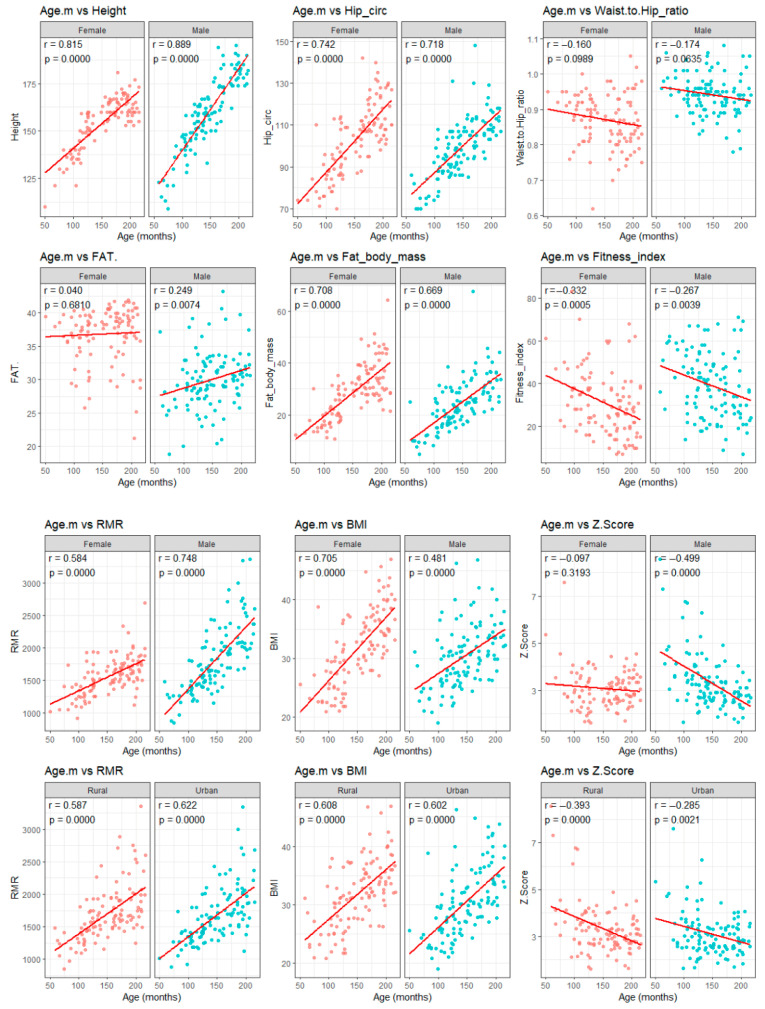
Correlation plots between age and anthropometric parameters (height, waist-to-hip ratio, BMI, and RMR).

Overall, these findings confirm the hypothesis that RMR increases with the degree of obesity, while emphasizing the need for more precise methods, such as indirect calorimetry, to accurately assess individual energy requirements and to guide personalized nutritional interventions in children and adolescents with obesity. The correlation plots illustrated in Figure 8 present the developmental trends observed in our cohort. As shown in the figures, age displayed strong positive associations with height, hip circumference, FM, BMI, and RMR across both sexes and residential environment. These consistent upward trajectories likely reflect normal growth processes combined with the progressive accumulation of adiposity seen in pediatric obesity. The strong correlation between age and RMR supports the notion that metabolic demand rises substantially during late childhood and adolescence. Interestingly, waist-to-hip ratio did not vary significantly with age, suggesting that patterns of fat distribution remained relatively stable across age groups. These findings align with previous reports indicating that central adiposity in children may be influenced more by adiposity level than by chronological age. The fitness index decreased significantly with age in both females (r = −0.332, *p* < 0.001) and males (r = −0.267, *p* = 0.004), which may reflect reduced physical activity levels or diminishing cardiorespiratory fitness during adolescence. This trend is clinically relevant as patients with obesity have lower fitness levels and have been linked to worsening metabolic outcomes in youth.

The inverse relationship between age and BMI z-score (r = −0.393, *p* < 0.001), especially prominent in males and in rural participants, suggests that although BMI increases with age, adolescents may fall into lower percentile categories relative to age and sex reference populations. This pattern reinforces the importance of interpreting BMI within an age-standardized framework.

These results support the utility of integrating anthropometric evaluation with energetic metabolism assessment using IC, correlated with functional indicators and body composition. These findings underscore the need for age-specific and appropriate assessment tools when evaluating growth, body composition, and metabolic risk in children and adolescents with obesity.

A broad biochemical and hematological profile was assessed in the study cohort. Correlation coefficients and corresponding *p*-values for all biochemical parameters, including fasting glucose and lipid profile, are detailed in Supplementary Material, Tables S1–S3. Hematological parameters were within expected pediatric ranges. Hemoglobin levels varied between 9.1 and 17.3 g/dL, with a mean of 13.48 ± 1.25 g/dL, and hematocrit ranged from 32.0 to 49.6% (mean 40.39 ± 3.18%). According to the values, nine patients were diagnosed with anemia. Platelet counts demonstrated substantial variability (165,000–562,000/µL), with a mean of 326.89 ± 60.58/µL, but within expected pediatric ranges.

Inflammatory markers showed moderate dispersion, with C-reactive protein (CRP) ranging from 0.10 to 69.02 mg/L (mean 4.40 ± 6.65 mg/L) and erythrocyte sedimentation rate (VSH) ranging from 2 to 70 mm/h (mean 11.63 ± 10.20 mm/h).

Liver enzymes showed wide ranges. Alanine aminotransferase (ALT/GPT) ranged from 7 to 226 U/L (mean 28.62 ± 23.29 U/L), while aspartate aminotransferase (AST/GOT) ranged between 9 and 143 U/L (mean 26.07 ± 13.12 U/L). Gamma-glutamyl transferase (GGT) presented a mean of 30.78 ± 12.55 U/L. The AST/ALT ratio had a mean of 1.11 ± 0.52.

Metabolic markers indicated considerable variability. Fasting plasma glucose ranged from 58 to 328 mg/dL (mean 86.25 ± 19.42 mg/dL), while glycated hemoglobin (HbA1c) varied between 2.7% and 11.2%. Insulin values (*n* = 96) showed wide dispersion (2.40–129.40 µU/mL, mean 19.78 ± 15.83 µU/mL), while the homeostatic model assessment of insulin resistance (HOMA-IR index, *n* = 96) ranged from 0.60 to 25.25 (mean 4.21 ± 3.26), indicating that a substantial proportion of participants presented with insulin resistance.

The lipid profile demonstrated elevated triglycerides with broader variability of 100.81 ± 49.99 mg/dL (range 25–292 mg/dL). Total cholesterol ranged from 87 to 276 mg/dL (mean 161.96 ± 31.62 mg/dL), LDL cholesterol averaged 100.02 ± 31.03 mg/dL, HDL averaged 47.75 ± 11.16 mg/dL, and VLDL averaged 20.58 ± 10.68 mg/dL. Triglycerides/HDL cholesterol had a mean of 2.30 ± 1.51.

Renal function markers remained within normal pediatric limits: creatinine averaged 0.60 ± 0.14 mg/dL, and urea averaged 26.14 ± 5.66 mg/dL. Uric acid presented a mean of 5.18 ± 2.54 mg/dL. Electrolytes and mineral parameters were within expected intervals: total calcium averaged 9.86 ± 0.38 mg/dL, ionized calcium averaged 4.26 ± 0.21 mg/dL, and magnesium averaged 2.01 ± 0.29 mg/dL. Total serum proteins averaged 7.38 ± 0.46 g/dL. Vitamin D levels (*n* = 25) ranged from 5.3 to 80.10 ng/mL, with a mean of 20.04 ± 14.71 ng/mL, indicating a predominantly deficit profile.

Thyroid function markers showed normal mean thyroid-stimulating hormone (TSH) values (mean 2.09 ± 0.98 µIU/mL), while free thyroxine (freeT4) levels averaged 1.02 ± 0.36 ng/dL. Anti-thyroid peroxidase (anti-TPO) antibodies had a mean value of 1.87 ± 8.47, and cortisol concentrations (*n* = 76) showed a mean of 12.66 ± 5.93 μg/dL.

Hepatic steatosis was evaluated in the study lot, and a total of 75 children and adolescents (33.6%) presented steatosis, while 148 participants (66.4%) showed no signs of fatty liver disease. When stratified by sex, steatosis remained more frequent in males. Among boys, 41 patients had steatosis, compared with 34 girls. These results indicate a higher susceptibility to hepatic fat accumulation among male participants.

No significant correlations were observed between RMR and fasting glucose (r = 0.075, *p* = 0.267), total cholesterol (r = 0.002, *p* = 0.977), LDL cholesterol (r = −0.052, *p* = 0.440), HDL cholesterol (r = −0.008, *p* = 0.907), triglycerides (r = 0.008, *p* = 0.908), or VLDL cholesterol (r = 0.006, *p* = 0.928). Similarly, no significant correlations were found between RMR and ALT (r = −0.113, *p* = 0.093), AST (r = −0.053, *p* = 0.433), insulin (r = −0.023, *p* = 0.825), cortisol (r = −0.161, *p* = 0.164), TSH (r = 0.097, *p* = 0.147), and freeT4 (r = 0.050, *p* = 0.460). However, RMR showed weak correlation with the HbA1c (r = 0.138, *p* = 0.039), magnesium (r = 0.144, *p* = 0.031), and AST/ALT ratio (r = 0.185, *p* = 0.006), while the triglycerides/HDL cholesterol ratio was not significantly associated with RMR (r = −0.005, *p* = 0.938).

BMI showed weak positive correlation with platelet count (r = 0.154, *p* = 0.021) and negative correlations with urea (r = −0.150, *p* = 0.025), uric acid (r = −0.135, *p* = 0.044), and GGT (r = −0.155, *p* = 0.020).

## 4. Discussion

Childhood and adolescence are critical development periods characterized by increased vulnerability to obesity and its associated health complications, commonly driven by unhealthy dietary patterns and sedentary behavior [30,31,32]. Our study cohort of 223 children and adolescents mirrors this trend, highlighting characteristics commonly associated with excess weight in children and adolescents.

In the present study, assessment of RMR through IC provides valuable insight into the metabolic status of children with obesity, which is essential for nutritional evaluation. According to the study by Acar-Tek et al., predictive equations show considerable systematic bias relative to IC, with markedly lower accuracy and higher RMR values, especially across the pediatric population with obesity, underscoring their limited reliability in clinical practice [33]. Although these equations are more advantageous in terms of cost and ease of use, they are less precise than direct measurements because of the substantial variability in estimates when applied to individuals whose characteristics differ from the populations in which they were originally validated [34,35]. IC is widely regarded as a reference method for estimating RMR, but its limited accessibility in routine clinical settings has prompted the development of predictive models [34,36].

Inter-individuality variability in RMR has been observed in diverse cohorts. In pediatric populations with obesity, measured RMR often shows wide distributions relative to predictive values even after adjustment for LM, underscoring individual differences in energy metabolism [37]. Similar variability patterns have been documented in adult cohorts, where sex, body composition, and obesity status contribute to RMR differences [22,38]. The actual study on 223 children and adolescents fits squarely within the high-risk category for metabolic health. Our data show that RMR, measured directly by IC, increases with age, body size, and FM in both sexes and geographic area. Such findings align with the recognized concept, from international studies, that metabolic demand increases with growth and physiological development, and this rise is particularly pronounced in children with obesity [37,39]. Moreover, metabolomic phenotyping approaches have identified distinct metabolic profiles within pediatric obesity, indicating heterogeneity beyond anthropometric measures such as BMI [40]. Studies in selected pediatric groups using IC further illustrate the value of direct RMR assessment in characterizing metabolic status [37,41]. In this context, the descriptive stratification applied in our cohort identified 32.3% of participants as having a slow RMR, 62.3% as normal, and 5.4% as fast, based on predefined thresholds applied to measured RMR values. In contrast, Tamini et al. reported 60.6% normometabolic, 25.5% hypermetabolic, and 13.9% hypometabolic children with obesity using IC and the estimated REE using the Molnar equation [19]. Abawi et al. observed 21% decreased and 24% elevated measured REE relative to predicted values [37]. These differences likely reflect non-equivalent classification methods and thresholds; therefore, our slow/normal/fast categories should be interpreted as descriptive within the cohort and should not be considered directly equivalent to hypo- or hypermetabolic phenotypes defined using measured and predicted RMR.

The wide range of RMR values observed in our cohort is consistent with previous pediatric studies using IC, which reported substantial inter-individual variability even within homogeneous obesity groups [37,42,43]. This observation aligns with previous reports indicating that higher RMR values in children with obesity are largely driven by increased body mass and LM, whereas weight-adjusted or LM-adjusted RMR may vary considerably among individuals [44]. Previous work by Zapata et al. showed that in children and adolescents, obesity does not appear to be driven by a reduction in RMR, as it is normalized to LM and does not differ significantly between normal weight, overweight, and obese groups [44]. These findings reinforce the need to interpret metabolic rate in relation to body composition rather than weight alone.

Overall, these findings indicate that resting metabolic rate is positively associated with the degree of adiposity in children and adolescents with obesity. The results also highlight the value of indirect calorimetry for obtaining individualized estimates of energy expenditure, which may support more accurate nutritional planning in clinical practice [45]. Our body composition findings, which were derived from calculations integrated into the calorimetry system and based on three standard skinfold measurements, provide an accessible and clinically relevant estimate of adiposity in our pediatric cohort. Skinfold-derived body composition is a widely used field method in children and adolescents, supported by established predictive equations such as those proposed by Slaughter at al., which were specifically validated in youth and remain among the accepted techniques for estimating FM at this age group [46,47]. Although skinfold measurements are inherently operator dependent and less precise than reference techniques such as dual-energy X-ray absorptiometry, there are studies that shows that they offer reasonably accurate approximations of body fat, particularly in large clinical samples where advanced imaging is not feasible. Recent studies have continued to evaluate the validity and comparability of skinfold-based body composition estimates with reference techniques. For example, Lopez-Gonzalez et al. compared skinfold thickness measurements with multiple reference methods, including DXA and air displacement plethysmography, in children and adolescents, highlighting differences across methodologies [47]. Similarly, van Beijsterveldt et al. developed and validated skinfold-based prediction equations for pediatric populations, demonstrating the ongoing refinement of anthropometric approaches [48]. These recent findings underscore the utility of skinfold measurements while cautioning about potential method-dependent bias and variability.

In this context, our use of skinfold-integrated calorimetric calculations represents an appropriate and literature-supported approach for characterizing body composition in children with overweight and obesity, allowing us to contextualize metabolic findings such as RMR within an established anthropometric framework. Also, the investigation adds meaningful value to patient follow-up assessments. The integration of skinfold-derived body composition estimates into calorimetric assessment represents a pragmatic and commonly used approach for characterizing adiposity in pediatric populations with overweight and obesity. This allows the resting metabolic rate to be interpreted within an established anthropometric framework. However, the present study was not designed to evaluate clinical outcomes or longitudinal follow-up, and any implications for patient monitoring should be considered exploratory.

Our cohort shows a high burden of adiposity and central fat distribution, accompanied by adverse functional and metabolic profiles. These findings align with contemporary guidance that frames pediatric obesity as a chronic, biologically complex disease that warrants proactive, comprehensive management, including behavioral therapy and, in selected cases, pharmacotherapy in adolescents.

Excess adiposity, especially central or visceral fat, has been associated with increased long-term cardiometabolic risk (insulin resistance, dyslipidemia, hypertension, and hepatic steatosis) [49]. In line with such evidence, a substantial proportion of our participants exhibited elevated systolic or diastolic blood pressure percentiles.

The positive associations between RMR and BMI, waist circumferences, and FM% observed in our data are consistent with the well-established role of LM as the principal determinant of RMR in youth, while comparatively smaller effects are attributed to FM and sex or pubertal development. Our use of indirect calorimetry is consistent with previous studies, indicating that this method provides reproducible and clinically useful estimates of resting metabolic rate in adolescents with overweight and obesity [19,22,50,51].

Given the dispersion around the BMI–RMR regression, our results suggests that IC may be particularly useful when individualized estimation of energy expenditure is required (e.g., complex obesity and metabolic comorbidity) in selected clinical contexts. Given the substantial discrepancies between measured and predicted energy expenditure in children with obesity, using IC to guide dietary prescriptions may reduce the risk of both underfeeding and overfeeding, thereby improving weight management outcomes [19].

Waist circumference measures tracked closely with BMI in our sample are reinforcing their utility as practical indicators of cardiometabolic risk in pediatric populations. Anthropometric indicators such as BMI, waist circumference, and WHtR have been extensively evaluated in children and adolescents for their ability to identify individuals at elevated risk of cardiometabolic abnormalities [52]. In our cohort, WHtR was positively associated with RMR, although the magnitude of this association was modest. In contrast, a strong correlation was observed between WHtR and BMI, confirming that this ratio primarily reflects overall and central adiposity rather than serving as an independent determinant of RMR. Comparative evidence suggests that these measures perform similarly for risk screening in pediatric individuals, allowing clinicians and public health programs to select the most feasible option based on available resources and context. Studies have demonstrated that WHtR is a robust marker of central adiposity and is consistently associated with clusters of cardiometabolic risk factors such as dyslipidemia, elevated blood pressure, insulin resistance, and metabolic syndrome components across pediatric populations [52,53,54]. These findings affirm that waist circumference, waist-to-hip ratio, and WHtR provide complementary information to BMI and can be incorporated into comprehensive risk assessment strategies for children and adolescents with overweight and obesity.

The predominance of BP values in high percentiles we observed mirrors the strong link between obesity and youth hypertension and underscores the importance of systematic screening and staged management set out in the American Academy of Pediatrics guideline [27]. Accurate BP measurement, ambulatory confirmation when indicated, and lifestyle therapy are emphasized. Indeed, the link between obesity and pediatric hypertension is well documented, and children with excess weight are at significantly higher risk of elevated blood pressure compared with their normal weight peers [53,55,56,57,58]. The high prevalence of abnormal BP percentiles in our cohort underlines the clinical importance of early screening for hypertension in pediatric obesity and not just for short-term health but for preventing long-term cardiovascular morbidity.

From a clinical and public health standpoint, our findings support the need for a comprehensive, multi-parametric assessment in children with obesity: anthropometry, body composition, RMR (measured not estimated), and biochemical/clinical markers (e.g., blood pressure, lipid profile, glucose, and liver enzymes). Such a multidisciplinary approach provides a more accurate metabolic profile, allowing tailored dietary and lifestyle interventions. Given that predictive equations for energy expenditure may misestimate true needs in obese children, using measured RMR can improve the precision of nutritional prescriptions and potentially enhance intervention efficacy [19]. With regard to biochemical parameters, RMR showed no meaningful associations with most markers of glucose and lipid metabolism, supporting the concept that basal energy expenditure in pediatric obesity is primarily driven by body size and composition rather than by circulating metabolic biomarkers. Only a weak association was observed with the AST/ALT ratio, while lipid-derived indices such as the triglyceride-to-HDL cholesterol ratio were not significantly related to RMR. Although most biochemical markers in our cohort did not show strong correlations with resting metabolic rate, previous pediatric studies have reported modest associations between REE and metabolic parameters such as leptin and markers of insulin sensitivity. For example, leptin concentrations have shown positive correlations with REE in children and adolescents, and indices of insulin resistance such as HOMA have exhibited weak but significant relationships with REE in pediatric cohorts [44,59]. These findings are consistent with previous observations that standard biochemical markers provide limited information on inter-individual variability in RMR in young populations and further emphasize the predominant role of anthropometric and body composition determinants [37].

Although statistically significant, the observed association with the fitness index was weak and should be interpreted cautiously as this parameter reflects a device-generated composite score rather than a direct measure of physical fitness. However, the inverse direction of the association is in line with broader syntheses reporting consistent relationships between higher cardiorespiratory fitness and adiposity, lipid profile, insulin resistance, vascular markers, mental health, and overall cardiometabolic risk in pediatric populations. Additionally, longitudinal and cohort studies indicate that higher levels of cardiorespiratory fitness and improvements in physical activity are associated with a lower risk of developing obesity and adverse cardiometabolic profiles and that elevated baseline CRF predicts more favorable trajectories for BMI and BP [60]. These findings support current recommendations to promote structured, developmentally appropriate physical activity as part of care pathways for young people with overweight and obesity [60,61].

From a clinical perspective, the integration of IC, anthropometry, body composition assessment, and biochemical markers offers a more comprehensive evaluation of children with obesity. Measured RMR allows for the development of more precise, individualized nutritional recommendations, which may improve adherence and treatment outcomes. However, our study has limitations because its cross-sectional design precludes causal inferences regarding the relationships between adiposity, metabolic rate, biochemical markers, and BP. The absence of an age- and genre-matched non-obese control group limits the ability to determine whether the observed resting metabolic rate values are obesity-specific or reflect characteristics of the local pediatric population. Therefore, the present findings should be interpreted as descriptive. To provide context, our results were benchmarked against published pediatric reference data derived from indirect calorimetry in non-obese populations. We also did not include longitudinal follow-up, which would be valuable to assess changes over time or the impact of interventions. The lack of data on socioeconomic status, diet, and physical activity represents an important limitation when interpreting variability in RMR and extrapolating the results to other settings.

Future research should focus on prospective, longitudinal studies that track changes in RMR, body composition, and metabolic markers over time, especially in response to individualized lifestyle or nutritional interventions. Such studies could clarify whether adjusting energy intake based on measured RMR leads to better weight control and reduced cardiometabolic risk. Interventional trials using calorimetry-guided dietary plans would help determine the clinical benefits of personalized energy prescription in pediatric obesity.

### Limitations

This study should be interpreted in light of several methodological limitations. Its cross-sectional, single-center design does not allow causal inference and may reduce the transferability of the results to different pediatric populations. The absence of an age- and sex-matched non-obese control group further limits the ability to determine whether the observed RMR values are specific to obesity or reflect local normative patterns. In addition, socioeconomic status, dietary intake, and habitual physical activity were not systematically assessed, which may contribute to unexplained inter-individual variability in RMR.

Although IC was performed under standardized conditions, RMR was not normalized to LM nor modeled against predicted values, which restricts direct comparability with metabolic phenotyping approaches used in large epidemiological cohorts. Furthermore, the stratification into slow, normal, and fast metabolic rate categories relied on age-adjusted thresholds provided by the calorimeter software rather than on externally validated criteria. Finally, the fitness index represents a proprietary composite score generated by the calorimeter software, and the exact calculation algorithm is not publicly disclosed by the manufacturer. Therefore, this score was analyzed as a descriptive, device-generated indicator rather than a validated standalone measure of physical fitness. The lack of transparency regarding its calculation limits the interpretation of its association with RMR.

## 5. Conclusions

This study demonstrates that excess adiposity in children and adolescents is closely associated with unfavorable metabolic, cardiovascular, and functional profiles. Central obesity was highly prevalent and strongly linked to elevated blood pressure, impaired fitness, and increased resting metabolic requirements. Although body mass index correlated with metabolic risk, waist-based indices and absolute fat mass provided more precise markers of cardiometabolic burden. The presence of hepatic steatosis and insulin resistance in a substantial proportion of participants further underscores the early onset of obesity-related complications.

These results support the use of integrated assessment based on anthropometry, laboratory markers, and functional testing, in order to improve risk stratification and to guide individualized interventions. These findings provide descriptive data on resting metabolic rate measured by indirect calorimetry using the Fitmate system in a regional pediatric obesity cohort and may inform future studies incorporating appropriate control groups. Early identification and targeted management, including structured physical activity and tailored nutritional therapy, remain essential to prevent progression toward long-term metabolic disease in pediatric populations with obesity.

## Figures and Tables

**Table 1:** Descriptive statistics.

**Statistics**																	
			**Age (Years)**	**Age (Months)**		**Weight (kg)**	**Height (cm)**		**BMI (kg/m^2^)**	**Z-Score**		**Abdominal Circumference (cm)**		**Hip Circumference (cm)**		**Waist Circumference (cm)**	
N	Valid		223	223		223	223		223	223		223		223		223	
	Missing		0	0		0	0		0	0		0		0		0	
Mean			12.03	149.65		79.7785	157.48		31.2154	3.2094		97.41		101.30		91.63	
Median			12.00	151.00		80.0000	159.00		30.9000	3.0600		98.00		103.00		91.00	
Std. Deviation			3.319	39.963		26.18737	16.576		5.84084	1.01461		14.729		15.362		13.911	
Variance			11.017	1597.039		685.778	274.755		34.115	1.029		216.936		235.993		193.513	
Minimum			4	50		25.00	109		19.00	1.61		56		70		52	
Maximum			17	216		156.60	195		46.90	8.56		148		148		140	
**Statistics**																	
					**RMR (kcal/Day)**			**Waist-to-Hip Ratio**			**FM%**		**FM (kg)**		**Systolic BP (mmHg)**		**Diastolic BP (mmHg)**
N		Valid			223			223			223		223		223		223
		Missing			0			0			0		0		0		0
Mean					1687.53			1.2796			33.261		26.751		118.30		81.68
Median					1632.00			0.9200			32.900		26.400		116.00		80.00
Std. Deviation					425.476			5.56481			5.3731		10.2120		14.746		11.037
Variance					181,029.565			30.967			28.870		104.284		217.447		121.822
Minimum					846			0.62			18.8		4.7		89		55
Maximum					3357			84.00			43.2		67.7		174		139

## Data Availability

The raw data supporting the conclusions of this article will be made available by the authors upon reasonable request, without undue restriction.

## Supplementary Materials

The following supporting information can be downloaded at https://www.mdpi.com/article/10.3390/diagnostics16020320/s1, Table S1: Descriptive Statistics of biochemical and hematological profile; Table S2: Pearson correlations coefficients between RMR, BMI, biochemical and hematological profile; Table S3: Pearson correlations coefficients between RMR, BMI, and biochemical profile.

## Abbreviations

The following abbreviations are used in this manuscript:

ALT/GPT	Alanine aminotransferase
Anti-TPO antibodies	Anti-thyroid peroxidase antibodies
AST/GOT	Aspartate aminotransferase
BMI	Body mass index
BP	Blood pressure
CRP	C-reactive protein
FM	Fat mass
FM%	Fat mass percentage
FreeT4	Free thyroxine
GGT	Gamma-glutamyl transferase
HbA1c	Glycated hemoglobin
HOMA-IR	Homeostatic model assessment of insulin resistance
IC	Indirect calorimetry
LM	Lean mass
MASLD	Metabolic dysfunction-associated steatotic liver disease
REE	Resting energy expenditure
RMR	Resting metabolic rate
RQ	Respiratory quotient
SD	Standard deviation
T2DM	Type 2 diabetes mellitus
TEE	Total energy expenditure
TSH	Thyroid-stimulating hormone
VO_2_	Oxygen consumption
VSH	Erythrocyte sedimentation rate
WC	Waist circumference
WHO	World Health Organization
WHtR	Waist-to-height ratio
