# Supplementary material for: Obesity and Resting Metabolic Rate Assessed by Indirect Calorimetry in Pediatric Patients from Northeastern Romania
Source: Diagnostics (Basel). 2026 Jan 19;16(2):320. doi: 10.3390/diagnostics16020320 (PMC12839848; doi:10.3390/diagnostics16020320)
Supplement: Supplementary file 1 [file diagnostics-16-00320-s001.zip › diagnostics-4074089-supplementary.pdf]

Supplementary material

Table S1. Descriptive statistics of biochemical and hematological profiles.

Descriptive Statistics

	N	Minimum	Maximum	Mean	Std. Deviation	Variance
Hemoglobin (g/dl)	223	9,10	17,30	13,4807	1,25179	1,567
Hematocrit (%)	223	32,00	49,60	40,3942	3,18182	10,124
Neutrophils (*10^3/uL)	223	,34	59,80	5,1243	5,69386	32,420
Lymphocytes (*10^3/uL)	223	1,17	23,50	2,8201	1,62701	2,647
Platelets (*10^3/uL)	223	165,00	562,00	326,8969	60,58798	3670,904
Serum iron (ug/dl)	223	14,00	174,00	69,7220	32,96304	1086,562
Erythrocyte sedimentation rate (mm/1h)	223	2,00	70,00	11,6265	10,19790	103,997
C-reactive protein (mg/L)	223	,10	69,52	4,4066	6,65638	44,307
Alanine aminotransferase (ALT/TGP) (U/L)	223	7,00	226,00	28,6233	23,29350	542,587
Aspartate aminotransferase (AST/TGO) (U/L)	223	9,00	143,00	26,0762	13,12616	172,296
Urea (mg/dl)	223	14,00	52,00	26,1390	5,66111	32,048
Creatinine (mg/dl)	223	,24	1,09	,5952	,14152	,020
Uric acid (mg/dl)	223	2,80	10,00	5,1804	2,54922	6,499
Glucose (mg/dL)	223	58,00	328,00	86,2466	19,42454	377,313
HbA1c (%)	223	2,70	11,20	5,4036	4,79392	22,982
Total cholesterol (mg/dl)	223	87,00	276,00	161,9552	31,61904	999,764
LDL-C (mg/dl)	223	23,00	221,00	100,0220	31,02937	962,822
VLDL-C (mg/dl)	223	5,00	67,00	20,5794	10,68541	114,178
HDL-C (mg/dl)	223	24,00	92,00	47,7525	11,15525	124,440
Triglycerides (mg/dl)	223	25,00	292,00	100,0807	49,99345	2499,345
Gamma-glutamyl transferase (U/L)	223	7,00	127,00	30,7892	12,55135	157,536
Total calcium (mg/dl)	223	8,90	10,80	9,8600	,38012	,144
Ionized calcium (mg/dl)	223	3,33	4,85	4,2571	,21112	,045
Magnesium (mg/dl)	223	1,52	4,95	2,0113	,29495	,087
Total protein (g/l)	223	62,90	94,80	73,9830	4,46031	19,894
25-OH vitamin D (ng/ml)	25	5,30	80,10	20,0400	14,71289	216,469
Thyroid-stimulating hormone (TSH) (μUI/mL)	223	,37	8,20	2,0965	,98589	,972
Free thyroxine (FreeT4) (ng/dL)	223	,10	5,91	1,0225	,36090	,130

Anti-TPO antibodies (UI/ml)	66	,00	64,07	1,8708	8,46641	71,680
Insulin (uUI/ml)	96	2,40	129,40	19,7784	15,83437	250,727
Cortisol (μg/dL)	76	4,50	31,73	12,6646	5,93233	35,193
HOMA-insulin resistance score	96	,60	25,25	4,2100	3,25581	10,600
Valid N (listwise)	1					

Tabel S2. Pearson correlations coefficients between RMR, BMI, and biochemical and hematological profiles.

Correlations

		RMR (kcal/day)	BMI (kg/m^2)	Hemoglobin (g/dl)	Hematocrit (%)	Neutrophils (*10^3/uL)	Lymphocytes (*10^3/uL)	Platelets (*10^3/uL)	Platelet Distribution Width (fl)	Serum Iron (ug/dl)	Erythrocyte Sedimentation Rate (mm/1h)	C-reactive protein (mg/L)	Alanine Aminotransferase (ALT/TGP) (U/L)	Aspartate Aminotransferase (AST/TGO) (U/L)	AST:ALT Ratio (TGO/TGP)	Urea (mg/dl)	Creatinine (mg/dl)	Uric Acid (mg/dl)	Glucose (mg/dL)	HbA1c (%)	Total Cholesterol (mg/dl)
RMR (kcal/day)	Pearson correlation	1	,515	-,059	-,054	-,080	-,094	,030	-,099	,003	-,048	-,095	-,113	-,053	,185	-,086	-,052	-,109	,075	,138	,002
	Sig. (2-tailed)		,000	,381	,426	,236	,162	,656	,141	,967	,475	,159	,093	,433	,006	,203	,442	,104	,267	,039	,977
	N	223	223	223	223	223	223	223	223	223	223	223	223	223	223	223	223	223	223	223	223
BMI (kg/m^2)	Pearson correlation	,515	1	-,051	-,036	-,031	,037	,154	-,061	-,018	,085	-,008	-,050	-,058	,028	-,150	,005	-,135	,050	,030	-,020

	Sig. (2-tailed)	,000		,448	,590	,641	,588	,021	,365	,784	,204	,902	,458	,385	,674	,025	,936	,044	,458	,660	,769
	N	223	223	223	223	223	223	223	223	223	223	223	223	223	223	223	223	223	223	223	223
Hemoglobin (g/dl)	Pearson correlation	-,059	-,051	1	,874	-,059	-,057	-,326	,136	,357	-,233	-,119	,205	,058	-,181	,112	,302	,087	,190	,021	,059
	Sig. (2-tailed)	,381	,448		,000	,383	,397	,000	,042	,000	,000	,077	,002	,390	,007	,094	,000	,193	,004	,759	,383
	N	223	223	223	223	223	223	223	223	223	223	223	223	223	223	223	223	223	223	223	223
Hematocrit (%)	Pearson correlation	-,054	-,036	,874	1	-,075	-,002	-,188	,177	,268	-,203	-,138	,194	,004	-,224	,148	,428	,080	,188	,011	,064
	Sig. (2-tailed)	,426	,590	,000		,266	,971	,005	,008	,000	,002	,040	,004	,955	,001	,027	,000	,233	,005	,872	,338
	N	223	223	223	223	223	223	223	223	223	223	223	223	223	223	223	223	223	223	223	223
Neutrophils ($*10^3$/uL)	Pearson correlation	-,080	-,031	-,059	-,075	1	,002	-,094	,228	-,062	,134	,113	,003	-,007	-,043	,030	,012	-,060	-,054	-,007	-,111
	Sig. (2-tailed)	,236	,641	,383	,266		,978	,162	,001	,356	,045	,093	,966	,912	,524	,652	,853	,371	,422	,922	,098
	N	223	223	223	223	223	223	223	223	223	223	223	223	223	223	223	223	223	223	223	223

Lymphocytes (*10^3/uL)	Pearson correlation	-,094	,037	-,057	-,002	,002	1	,206	-,131	-,058	,026	,039	-,008	,017	,023	,091	-,074	-,050	-,006	-,023	,049
	Sig. (2-tailed)	,162	,588	,397	,971	,978		,002	,050	,386	,700	,560	,907	,801	,731	,174	,269	,458	,931	,729	,464
	N	223	223	223	223	223	223	223	223	223	223	223	223	223	223	223	223	223	223	223	223
Platelets (*10^3/uL)	Pearson correlation	,030	,154	-,326	-,188	-,094	,206	1	-,375	-,233	,113	-,036	,048	,076	,022	-,121	-,168	-,051	-,058	-,025	,032
	Sig. (2-tailed)	,656	,021	,000	,005	,162	,002		,000	,000	,092	,590	,479	,258	,742	,072	,012	,450	,387	,709	,631
	N	223	223	223	223	223	223	223	223	223	223	223	223	223	223	223	223	223	223	223	223
Platelet distribution width (fl)	Pearson correlation	-,099	-,061	,136	,177	,228	-,131	-,375	1	,040	-,005	,035	,000	-,097	-,159	-,024	,215	-,005	-,039	-,052	-,112
	Sig. (2-tailed)	,141	,365	,042	,008	,001	,050	,000		,547	,941	,598	,997	,147	,017	,723	,001	,944	,560	,441	,094
	N	223	223	223	223	223	223	223	223	223	223	223	223	223	223	223	223	223	223	223	223
Serum iron (ug/dl)	Pearson correlation	,003	-,018	,357	,268	-,062	-,058	-,233	,040	1	-,046	-,123	,090	,065	-,075	,088	,123	,051	,180	,007	,097
	Sig. (2-tailed)	,967	,784	,000	,000	,356	,386	,000	,547		,490	,067	,182	,333	,266	,193	,067	,448	,007	,921	,148

	N	223	223	223	223	223	223	223	223	223	223	223	223	223	223	223	223	223	223	223	223
Erythrocyte sedimentation rate (mm/1h)	Pearson correlation	-,048	,085	-,233	-,203	,134	,026	,113	-,005	-,046	1	,122	-,064	-,043	-,015	,020	-,025	,014	-,105	-,041	,110
	Sig. (2-tailed)	,475	,204	,000	,002	,045	,700	,092	,941	,490		,069	,338	,525	,827	,762	,715	,831	,117	,539	,102
	N	223	223	223	223	223	223	223	223	223	223	223	223	223	223	223	223	223	223	223	223
C-reactive protein (mg/L)	Pearson correlation	-,095	-,008	-,119	-,138	,113	,039	-,036	,035	-,123	,122	1	-,034	-,042	-,032	,001	,003	-,021	-,099	-,010	,151
	Sig. (2-tailed)	,159	,902	,077	,040	,093	,560	,590	,598	,067	,069		,609	,532	,633	,991	,959	,755	,140	,885	,024
	N	223	223	223	223	223	223	223	223	223	223	223	223	223	223	223	223	223	223	223	223
Alanine aminotransferase (ALT/TGP) (U/L)	Pearson correlation	-,113	-,050	,205	,194	,003	-,008	,048	,000	,090	-,064	-,034	1	,780	-,473	,125	,003	,025	,102	,055	,101
	Sig. (2-tailed)	,093	,458	,002	,004	,966	,907	,479	,997	,182	,338	,609		,000	,000	,062	,969	,709	,127	,410	,132
	N	223	223	223	223	223	223	223	223	223	223	223	223	223	223	223	223	223	223	223	223
Aspartate aminotransferase	Pearson correlation	-,053	-,058	,058	,004	-,007	,017	,076	-,097	,065	-,043	-,042	,780	1	,044	,080	-,159	-,059	,008	,073	,078

(AST/TGO) (U/L)	Sig. (2-tailed)	,433	,385	,390	,955	,912	,801	,258	,147	,333	,525	,532	,000		,510	,237	,018	,377	,905	,279	,249
	N	223	223	223	223	223	223	223	223	223	223	223	223	223	223	223	223	223	223	223	223
AST:ALT ratio (TGO/TGP)	Pearson correlation	,185	,028	-,181	-,224	-,043	,023	,022	-,159	-,075	-,015	-,032	-,473	,044	1	-,122	-,128	-,115	-,035	-,033	-,056
	Sig. (2-tailed)	,006	,674	,007	,001	,524	,731	,742	,017	,266	,827	,633	,000	,510		,069	,056	,087	,602	,623	,406
	N	223	223	223	223	223	223	223	223	223	223	223	223	223	223	223	223	223	223	223	223
Urea (mg/dl)	Pearson correlation	-,086	-,150	,112	,148	,030	,091	-,121	-,024	,088	,020	,001	,125	,080	-,122	1	,088	,012	,148	,061	,074
	Sig. (2-tailed)	,203	,025	,094	,027	,652	,174	,072	,723	,193	,762	,991	,062	,237	,069		,188	,859	,027	,364	,268
	N	223	223	223	223	223	223	223	223	223	223	223	223	223	223	223	223	223	223	223	223
Creatinine (mg/dl)	Pearson correlation	-,052	,005	,302	,428	,012	-,074	-,168	,215	,123	-,025	,003	,003	-,159	-,128	,088	1	,132	,177	-,039	-,040
	Sig. (2-tailed)	,442	,936	,000	,000	,853	,269	,012	,001	,067	,715	,959	,969	,018	,056	,188		,048	,008	,561	,549
	N	223	223	223	223	223	223	223	223	223	223	223	223	223	223	223	223	223	223	223	223

Uric acid (mg/dl)	Pearson correlation	-,109	-,135	,087	,080	-,060	-,050	-,051	-,005	,051	,014	-,021	,025	-,059	-,115	,012	,132	1	,033	,020	,021
	Sig. (2-tailed)	,104	,044	,193	,233	,371	,458	,450	,944	,448	,831	,755	,709	,377	,087	,859	,048		,626	,767	,750
	N	223	223	223	223	223	223	223	223	223	223	223	223	223	223	223	223	223	223	223	223
Glucose (mg/dL)	Pearson correlation	,075	,050	,190	,188	-,054	-,006	-,058	-,039	,180	-,105	-,099	,102	,008	-,035	,148	,177	,033	1	,063	,046
	Sig. (2-tailed)	,267	,458	,004	,005	,422	,931	,387	,560	,007	,117	,140	,127	,905	,602	,027	,008	,626		,353	,493
	N	223	223	223	223	223	223	223	223	223	223	223	223	223	223	223	223	223	223	223	223
HbA1c (%)	Pearson correlation	,138	,030	,021	,011	-,007	-,023	-,025	-,052	,007	-,041	-,010	,055	,073	-,033	,061	-,039	,020	,063	1	,024
	Sig. (2-tailed)	,039	,660	,759	,872	,922	,729	,709	,441	,921	,539	,885	,410	,279	,623	,364	,561	,767	,353		,720
	N	223	223	223	223	223	223	223	223	223	223	223	223	223	223	223	223	223	223	223	223
Total cholesterol (mg/dl)	Pearson correlation	,002	-,020	,059	,064	-,111	,049	,032	-,112	,097	,110	,151	,101	,078	-,056	,074	-,040	,021	,046	,024	1
	Sig. (2-tailed)	,977	,769	,383	,338	,098	,464	,631	,094	,148	,102	,024	,132	,249	,406	,268	,549	,750	,493	,720	

N	223	223	223	223	223	223	223	223	223	223	223	223	223	223	223	223	223	223	223	223

Tabel S3. Pearson correlations coefficients between RMR, BMI, and biochemical profile.

Correlations

		RMR (kcal/day)	BMI (kg/m^2)	LDL-C (mg/dl)	VLDL-C (mg/dl)	HDL-C (mg/dl)	Triglycerides (mg/dl)	TG:HDL-C Ratio	Gamma-Glutamyl Transferase (U/L)	Total Calcium (mg/dl)	Ionized Calcium (mg/dl)	Magnesium (mg/dl)	Total Protein (g/l)	25-OH Vitamin D (ng/ml)	TSH (µUI/mL)	Free T4 (ng/dL)	Anti-TPO Antibodies (UI/ml)	Insulin (uUI/ml)	Cortisol (µg/dL)	HOMA-Insulin Resistance Score
RMR (kcal/day)	Pearson correlation	1	,515	-,052	,006	-,008	,008	-,005	,010	,040	,039	,144	,013	,178	,097	,050	-,016	-,023	-,161	,013
	Sig. (2-tailed)		,000	,440	,928	,908	,907	,938	,877	,557	,567	,031	,851	,395	,147	,460	,898	,825	,164	,902
	N	223	223	223	223	223	223	223	223	223	223	223	223	25	223	223	66	96	76	96
BMI (kg/m^2)	Pearson correlation	,515	1	-,018	,006	-,122	,003	,019	-,155	-,030	-,041	-,092	-,026	,042	-,027	,031	-,026	,124	,164	,173
	Sig. (2-tailed)	,000		,787	,923	,068	,964	,776	,020	,657	,539	,169	,694	,841	,690	,646	,834	,227	,156	,092
	N	223	223	223	223	223	223	223	223	223	223	223	223	25	223	223	66	96	76	96
LDL-C (mg/dl)	Pearson correlation	-,052	-,018	1	,233	-,072	,300	,216	,102	-,009	-,096	-,001	,210	-,144	,021	,064	-,123	,130	-,041	,099

	Sig. (2-tailed)	,440	,787		,000	,285	,000	,001	,131	,893	,152	,984	,002	,491	,756	,344	,323	,207	,726	,337
	N	223	223	223	223	223	223	223	223	223	223	223	223	25	223	223	66	96	76	96
VLDL-C (mg/dl)	Pearson correlation	,006	,006	,233	1	-,281	,900	,822	,191	,090	-,122	-,050	,183	-,231	,160	-,052	-,064	,447	-,123	,450
	Sig. (2-tailed)	,928	,923	,000		,000	,000	,000	,004	,182	,068	,454	,006	,267	,017	,441	,609	,000	,289	,000
	N	223	223	223	223	223	223	223	223	223	223	223	223	25	223	223	66	96	76	96
HDL-C (mg/dl)	Pearson correlation	-,008	-,122	-,072	-,281	1	-,342	-,588	-,077	,055	,116	-,010	-,150	-,226	-,014	-,048	,180	-,060	-,020	-,065
	Sig. (2-tailed)	,908	,068	,285	,000		,000	,000	,251	,415	,084	,880	,025	,278	,836	,478	,147	,562	,861	,527
	N	223	223	223	223	223	223	223	223	223	223	223	223	25	223	223	66	96	76	96
Triglycerides (mg/dl)	Pearson correlation	,008	,003	,300	,900	-,342	1	,925	,194	,107	-,134	-,048	,217	-,318	,176	-,062	-,060	,152	-,134	,179
	Sig. (2-tailed)	,907	,964	,000	,000	,000		,000	,004	,110	,046	,474	,001	,121	,008	,357	,634	,140	,247	,080
	N	223	223	223	223	223	223	223	223	223	223	223	223	25	223	223	66	96	76	96
TG:HDL-C ratio	Pearson correlation	-,005	,019	,216	,822	-,588	,925	1	,213	,064	-,148	-,036	,210	-,207	,141	-,032	-,082	,156	-,120	,173
	Sig. (2-tailed)	,938	,776	,001	,000	,000	,000		,001	,338	,027	,591	,002	,320	,036	,631	,514	,128	,304	,092
	N	223	223	223	223	223	223	223	223	223	223	223	223	25	223	223	66	96	76	96

Gamma-glutamyl transferase (U/L)	Pearson correlation	,010	-,155	,102	,191	-,077	,194	,213	1	,109	,002	,040	,094	,476	,108	-,036	-,119	,112	-,093	,095
	Sig. (2-tailed)	,877	,020	,131	,004	,251	,004	,001		,105	,978	,550	,164	,016	,109	,598	,342	,276	,423	,358
	N	223	223	223	223	223	223	223	223	223	223	223	223	25	223	223	66	96	76	96
Total calcium (mg/dl)	Pearson correlation	,040	-,030	-,009	,090	,055	,107	,064	,109	1	,494	,112	,289	,060	,151	-,025	,136	-,071	-,142	-,080
	Sig. (2-tailed)	,557	,657	,893	,182	,415	,110	,338	,105		,000	,096	,000	,774	,024	,706	,277	,492	,222	,438
	N	223	223	223	223	223	223	223	223	223	223	223	223	25	223	223	66	96	76	96
Ionized calcium (mg/dl)	Pearson correlation	,039	-,041	-,096	-,122	,116	-,134	-,148	,002	,494	1	,068	-,306	,004	-,038	,052	,104	,003	-,200	-,037
	Sig. (2-tailed)	,567	,539	,152	,068	,084	,046	,027	,978	,000		,309	,000	,984	,570	,436	,405	,979	,083	,717
	N	223	223	223	223	223	223	223	223	223	223	223	223	25	223	223	66	96	76	96
Magnesium (mg/dl)	Pearson correlation	,144	-,092	-,001	-,050	-,010	-,048	-,036	,040	,112	,068	1	,103	-,002	,150	,076	,031	-,101	-,049	-,135
	Sig. (2-tailed)	,031	,169	,984	,454	,880	,474	,591	,550	,096	,309		,127	,992	,025	,260	,805	,329	,676	,190
	N	223	223	223	223	223	223	223	223	223	223	223	223	25	223	223	66	96	76	96
Total protein (g/l)	Pearson correlation	,013	-,026	,210	,183	-,150	,217	,210	,094	,289	-,306	,103	1	,213	,122	-,084	,072	-,019	,050	-,040

	Sig. (2-tailed)	,851	,694	,002	,006	,025	,001	,002	,164	,000	,000	,127		,306	,069	,211	,563	,851	,668	,699
	N	223	223	223	223	223	223	223	223	223	223	223	223	25	223	223	66	96	76	96
25-OH vitamin D (ng/ml)	Pearson correlation	,178	,042	-,144	-,231	-,226	-,318	-,207	,476	,060	,004	-,002	,213	1	-,056	,379	1,000	-,290	-,065	-,349
	Sig. (2-tailed)	,395	,841	,491	,267	,278	,121	,320	,016	,774	,984	,992	,306		,792	,062	.	,275	,841	,203
	N	25	25	25	25	25	25	25	25	25	25	25	25	25	25	25	2	16	12	15
TSH (μUI/mL)	Pearson correlation	,097	-,027	,021	,160	-,014	,176	,141	,108	,151	-,038	,150	,122	-,056	1	,031	,034	-,092	-,145	-,103
	Sig. (2-tailed)	,147	,690	,756	,017	,836	,008	,036	,109	,024	,570	,025	,069	,792		,650	,788	,375	,212	,320
	N	223	223	223	223	223	223	223	223	223	223	223	223	25	223	223	66	96	76	96
Free T4 (ng/dL)	Pearson correlation	,050	,031	,064	-,052	-,048	-,062	-,032	-,036	-,025	,052	,076	-,084	,379	,031	1	-,079	,120	-,135	,105
	Sig. (2-tailed)	,460	,646	,344	,441	,478	,357	,631	,598	,706	,436	,260	,211	,062	,650		,529	,242	,244	,310
	N	223	223	223	223	223	223	223	223	223	223	223	223	25	223	223	66	96	76	96
Anti-TPO antibodies (UI/ml)	Pearson correlation	-,016	-,026	-,123	-,064	,180	-,060	-,082	-,119	,136	,104	,031	,072	1,000	,034	-,079	1	,075	-,119	,120
	Sig. (2-tailed)	,898	,834	,323	,609	,147	,634	,514	,342	,277	,405	,805	,563	.	,788	,529		,665	,562	,487
	N	66	66	66	66	66	66	66	66	66	66	66	66	2	66	66	66	36	26	36

Insulin (uUI/ml)	Pearson correlation	-,023	,124	,130	,447	-,060	,152	,156	,112	-,071	,003	-,101	-,019	-,290	-,092	,120	,075	1	,051	,974
	Sig. (2-tailed)	,825	,227	,207	,000	,562	,140	,128	,276	,492	,979	,329	,851	,275	,375	,242	,665		,671	,000
	N	96	96	96	96	96	96	96	96	96	96	96	96	16	96	96	36	96	73	95
Cortisol (µg/dL)	Pearson correlation	-,161	,164	-,041	-,123	-,020	-,134	-,120	-,093	-,142	-,200	-,049	,050	-,065	-,145	-,135	-,119	,051	1	,039
	Sig. (2-tailed)	,164	,156	,726	,289	,861	,247	,304	,423	,222	,083	,676	,668	,841	,212	,244	,562	,671		,743
	N	76	76	76	76	76	76	76	76	76	76	76	76	12	76	76	26	73	76	73
HOMA-insulin resistance score	Pearson correlation	,013	,173	,099	,450	-,065	,179	,173	,095	-,080	-,037	-,135	-,040	-,349	-,103	,105	,120	,974	,039	1
	Sig. (2-tailed)	,902	,092	,337	,000	,527	,080	,092	,358	,438	,717	,190	,699	,203	,320	,310	,487	,000	,743	
	N	96	96	96	96	96	96	96	96	96	96	96	96	15	96	96	36	95	73	96
